# Supplementary material for: Do Patients with Bronchiectasis Have an Increased Risk of Developing Lung Cancer? A Systematic Review
Source: Life (Basel). 2023 Feb 7;13(2):459. doi: 10.3390/life13020459 (PMC9961135; doi:10.3390/life13020459)
Supplement: Supplementary file 1 [file life-13-00459-s001.zip › S2 File Prospero protocol.pdf]

PROSPERO

International prospective register of systematic reviews

Bronchiectasis and Lung Cancer: a systematic review and meta-analysis

Citation

Nadia Castaldo, Alberto Fantin, Manera Massimiliano, Vincenzo Patruno, Giulia Sartori, Ernesto Crisafulli. Bronchiectasis and Lung Cancer: a systematic review and meta-analysis.

Review question

- Is there an association between adult noncystic fibrosis bronchiectasis (AB) and lung cancer (LC) risk?
- What is the overall prevalence of lung cancer LC in AB?
- What is the prevalence of LC in AB based on country?
- What is the prevalence of LC in AB based on gender?
- What is the prevalence of smoking in LC-AB patients?
- What is the prevalence of chronic obstructive pulmonary disease (COPD) in LC-AB patients?
- What is the prevalence of idiopathic pulmonary fibrosis (IPF) in LC-AB patients?
- What is the prevalence of asthma in LC-AB patients?
- What is the prevalence of non-IPD-intertitial lung diseases (ILD) in LC-AB patients?
- What is the prevalence of non-iatrogenic immunodeficiency in LC-AB patients? (non-iatrogenic immunodeficiency: HD ((haematologic disease (%)), HIV (%), CVID ((Common variable immune deficiency(%)), SIA ((Selective IgA deficiency(%)), others?
- What is the prevalence of iatrogenic immunodeficiency in LC-AB patients? (iatrogenic immunodeficiency: SOT ((solid organ transplant (%)), BMT ((bone marrow transplant(%)), AC ((autoimmune conditions(%)), others?
- What is the prevalence of chronic infections in LC-AB patients? (chronic infections :CPA ((chronic pulmonary aspergillosis(%)), MI ((mycobacterial infections(%)), RBP ((recurrent bacterial pneumonia (%)), others?)
- What is the prevalence of non-respiratory chronic disease in LC-AB patients? (non-respiratory chronic disease: CVD ((cardiovascular diseases (%)) , DM ((diabetes

mellitus (%)), CKD ((chronic kidney disease (%)), CLD ((chronic liver disease (%)), NC ((neurological conditions (%)), others)

- What is the prevalence of LC in AB according to clinical staging (I, II, II, IV)?
- What is the prevalence of the region and location of LC in LC-AB (region: RUL (right upper lobe), LUL (left upper lobe), RLL (right lower lobe), LLL (left lower lobe), upper, center, lower, and location: peripheral and central)?
- What is the prevalence of the region and location of AB in LC-AB (region: RUL (right upper lobe), LUL (left upper lobe), RLL (right lower lobe), LLL (left lower lobe), upper, center, lower, and location: peripheral and central)?
- What is the prevalence of the cellular (histological) subtypes of LC in AB ((ADC (adenocarcinoma (%)), SQCC (squamous-cell carcinoma (%)), SmCC (small-cell carcinoma (%)), LCC (large-cell carcinoma (%)), ADSQC (adeno-squamous carcinoma (%)), and others)?
- What is the prevalence of synchronous cancers with LC in AB (synchronous cancers: DSC ((digestive system cancers (%)), BC (breast cancer (%)), CTC (connective tissues cancer (%)), BC (brain cancers (%)), URUD (upper respiratory and upper digestive tracts (%)), and others)?
- What is the overall mortality of LC-AB patients?

Searches

The present study will be carried out in accordance with the Meta-analysis of Observational Studies in Epidemiology guideline, and according to the PRISMA statement.

Search strategy:

- A comprenhensive literature research was conducted through international databases, such as PubMed/Medline, Scopus, WebOfScience , OVID, and COCHRANE Library. Articles regarding lung cancer and bronchiectasis published before June, 21,2022, were searched using standard keywords as well as Mesh and Mesh Entry.

In addition, a manual search was also carried out independently by two researchers, in order to select relevant articles included in the reference lists of previously identified manuscripts.

Types of study to be included

Retrospective and prospective studies investigating on LC occurrence in bronchiectasis. The following exclusion criteria were used: (1) letters, editorials, expert opinions, case reports or case series with less than 10 patients, and reviews; (2) nonhuman studies; (3) duplicated or overlapped data.

Condition or domain being studied

Lung cancer in bronchiectasis.

Participants/population

Cohort, retrospective and prospective studies which have investigated LC in AB patients.

Intervention(s), exposure(s)

None.

Comparator(s)/control

Prevalence of LC in patients with AB will be compared with rates of non-occurrence of LC.

Context

Primary outcome(s)

Overall prevalence of lung cancer (LC) in adult noncystic fibrosis bronchiectasis (AB).

Secondary outcome(s)

Association between adult noncystic fibrosis bronchiectasis (AB) and lung cancer (LC) risk
Overall prevalence of lung cancer LC in AB
Prevalence of LC in AB based on country
Prevalence of LC in AB based on gender
Prevalence of smoking in LC-AB patients
Prevalence of chronic obstructive pulmonary disease (COPD) in LC-AB patients
Prevalence of idiopathic pulmonary fibrosis (IPF) in LC-AB patients
Prevalence of asthma in LC-AB patients
Prevalence of non-IPD-intertitial lung diseases (ILD) in LC-AB patients
Prevalence of non-iatrogenic immunodeficiency in LC-AB patients
Prevalence of iatrogenic immunodeficiency in LC-AB patients

Prevalence of chronic infections in LC-AB patients

Prevalence of non-respiratory chronic disease in LC-AB patients

Prevalence of LC in AB according to clinical staging (I, II, II, IV)

Prevalence of the region and location of LC in LC-AB

Prevalence of the region and location of AB in LC-AB

Prevalence of the cellular (histological) subtypes of LC in AB

Prevalence of synchronous cancers with LC in AB

Mortality of LC-AB patients

Data extraction (selection and coding)

The following information will be extracted and recorded into an Excel spreadsheet: authors, year, place, sample size (SS), periods of time, SS of AB , SS of LC, prevalence (LC in AB), cellular (histological) subtypes of LC and non-lung cancers, clinical staging, age (mean±SD), prevalence by gender, prevalence by smoking status, prevalence of respiratory and non-respiratory comorbidities, prevalence of immunodeficiency, prevalence of chronic infections, region and location of LC and AB .

Risk of bias (quality) assessment

After selecting relevant studies, the quality of the final studies elected for inclusion will be examined. The Newcastle-Ottawa Scale (NOS) checklist will be used. The scale includes eight sections, and classifies the studies with a scale from 0 to 8 from poor to high quality, respectively.

Strategy for data synthesis

The present study will be carried out in accordance with the Meta-analysis of Observational Studies in Epidemiology guideline, and according to the PRISMA guidelines. Five steps will be followed: the design and search strategy; the collection of articles and their systematic review; the assessment of the inclusion and exclusion criteria; and the qualitative assessment and statistical analysis of the data. All stages will be carried out independently by two researchers.

Data analysis will be performed using IBM SPSS Statistics v 20 (IBM Corp. Released 2011. IBM SPSS Statistics for Windows, Version 20.0. Armonk, NY: IBM Corp.), and the significance level of the test will be < 0.05.

Analysis of subgroups or subsets

As described above, we will perform subgroup analyses regarding LC occurrence and AB in different subsets of patients.   Furthermore, if sufficient data are available, we will perform the following subgroup analyses:

1. type of cancer (e.g. primary or metastatic lung cancer);

2. diagnosis (e.g. symptomatic patient, instrumental finding);

3. inflammation status (e.g. levels of serum C reactive protein, erythrocyte sedimentation rate, total white blood cell count, immunoglobulin levels);

4. pulmonary function measures before cancer diagnosis (e.g. FEV1,FVC, DLCO);

Contact details for further information

Alberto Fantin, MD

alberto.fantin@asufc.sanita.fvg.it

Organisational affiliation of the review

Department of Pulmonology, University Hospital of Udine (ASUFC), Udine, Italy.

Collaborators

Nadia Castaldo, Manera Massimiliano, Vincenzo Patruno, Giulia Sartori, Ernesto Crisafulli

Anticipated or actual start date

6/9/2022

Anticipated completion date

15/12/2022

Funding sources/sponsors

None.

Conflicts of interest

None.

Language

English.

Country

Italy .

Stage of review

Review_Ongoing

Subject index terms status

Subject indexing assigned by CRD

Subject index terms

Humans; Adult noncystic fibrosis bronchiectasis; Incidence; Prevalence; Lung; Lung Neoplasms; Risk Factors

Date of registration in PROSPERO

25/09/2022

Date of publication of this version

15/11/2022

Stage	Started	Completed
Preliminary searches	Yes	Yes
Piloting of the study selection process	Yes	Yes
Formal screening of search results against eligibility criteria	Yes	Yes
Data extraction	Yes	Yes
Risk of bias (quality) assessment	Yes	Yes
Data analysis	Yes	Yes
