# Supplementary material for: Do Patients with Bronchiectasis Have an Increased Risk of Developing Lung Cancer? A Systematic Review
Source: Life (Basel). 2023 Feb 7;13(2):459. doi: 10.3390/life13020459 (PMC9961135; doi:10.3390/life13020459)
Supplement: Supplementary file 1 [file life-13-00459-s001.zip › Table S1- search strategy.pdf]

Table S1 . Search strategy used for one of the databases

(Accessed by using University of Udine - VPN). Search performed on: Sept, 06,2022

Databases	Group	Search Syntax	Records
PubMed/Medline	1	(“Bronchiectasis”[MeSH Terms] OR “Bronchiectasis”[Title/Abstract])	**15.120**
	2	(“Lung Neoplasms”[MeSH Terms] OR “Pulmonary Neoplasms”[Title/Abstract] OR “Lung Neoplasm”[Title/Abstract] OR “Pulmonary Neoplasm”[Title/Abstract] OR “Lung Cancer”[Title/Abstract] OR “Lung Cancers”[Title/Abstract] OR “Pulmonary Cancer”[Title/Abstract] OR “Pulmonary Cancers”[Title/Abstract] OR “Cancer of the Lung”[Title/Abstract] OR “Cancer of Lung”[Title/Abstract])	**325.198**
	**#1 AND #2**		**973**
Scopus	1	TITLE-ABS-KEY(“Bronchiectasis”)	**23.661**
	2	TITLE-ABS-KEY (“Lung Neoplasms” OR “Pulmonary Neoplasms” OR “Lung Neoplasm” OR “Pulmonary Neoplasm” OR “Lung Cancer” OR “Lung Cancers” OR “Pulmonary Cancer” OR “Pulmonary Cancers” OR “Cancer of the Lung” OR “Cancer of Lung”)	**388.157**
	**#1 AND #2**		**1.576**
Web of Sciences	1	**TOPIC:**((“Bronchiectasis”))	**58**
	2	**TOPIC:** ((“Lung Neoplasms” OR “Pulmonary Neoplasms” OR “Lung Neoplasm” OR “Pulmonary Neoplasm” OR “Lung Cancer” OR “Lung Cancers” OR “Pulmonary Cancer” OR “Pulmonary Cancers” OR “Cancer of the Lung” OR “Cancer of Lung”))	**1.427**
	**#1 AND #2**		**1**
OVID (All resources)	1	Bronchiectasis.sh. or Bronchiectasis.af.	**41.875**
	2	Lung Neoplasms.sh. or Pulmonary Neoplasms.af. or Lung Neoplasm.af. or Pulmonary Neoplasm.af. or Lung Cancer.af. or Lung Cancers.af. or Pulmonary Cancer.af. or Pulmonary Cancers.af. or Cancer of the Lung.af. or Cancer of Lung.af.	**680.264**
	**#1 AND #2**		**4761**
COCHRANE		'("Bronchiectasis") in Title, Abstract, Keywords in Trials and Other Reviews and Cochrane Reviews'	**1.359**
		'("Lung Neoplasms" OR "Pulmonary Neoplasms" OR "Lung Neoplasm" OR "Pulmonary Neoplasm" OR "Lung Cancer" OR "Lung Cancers" OR "Pulmonary Cancer" OR "Pulmonary Cancers" OR "Cancer of the Lung" OR "Cancer of Lung") in Title, Abstract, Keywords in Trials and Other Reviews and Cochrane Reviews'	**23.794**
	**#1 AND #2**		**21**

	**Total evaluated**	**7732**
