# Supplementary material for: Do Patients with Bronchiectasis Have an Increased Risk of Developing Lung Cancer? A Systematic Review
Source: Life (Basel). 2023 Feb 7;13(2):459. doi: 10.3390/life13020459 (PMC9961135; doi:10.3390/life13020459)
Supplement: Supplementary file 1 [file life-13-00459-s001.zip › Table S2- Results corretta.pdf]

**Table S2. Major findings in literature and summary of results**

| Citation | Design, country | Participants, setting | Outcomes and primary objectives of the studies | N of patients with NCFB and LC | Incidence rates of LC in patients with NCFB (%) | Age ≥ 60 or ≥°65 , %(n) | Male sex, %,(n) | Ever smokers, %, (n) | COPD %, (n) | Major study results |
|---|---|---|---|---|---|---|---|---|---|---|
| **Kim Y et al. (2022) [1]** | Population-based cohort study, Korea | Patients from Korean NHIS database | The risk factors of lung cancer in participants with NCFB | 138 | 1.9 | 84.1 (116) | 79 (109) | 59.4 (82) | 35.5 (49) | Higher risk of incident LC in NCFB pts who were males (aHR = 3.54, 95% CI= 2.17–5.79) , and ever smokers ( aHR 3.10, [95% CI = 2.00–4.79] ).<br>Risk of LC higher in NCFB pts with CCI ≥2 (aHR 1.59 [95% CI 0.70–3.64]) |
| **Choi H et al. (2022) [2]** | Population-based cohort study, Korea | Patients from Korean NHIS database | The risk of lung cancer in participants with NCFB in comparison to those without NCFB | 1092 | 2.1 | 84.6 (924) | 73.4 (802) | 54.85 (599) | 55.2 (603) | Higher risk of LC in NCFB pts than non bronchiectasic ones (aHR 1.22 [95% CI, 1.14–1.30])<br>Risk of lung cancer higher in NCFB pts who smoked ≥ 20 pack/years (aHR 1.14 [95% CI, 1.03–1.26]), but not in those with a story of < 10 pack-years (aHR 1.23 [95% CI, 0.94–1.61]) and 10-19 pack/years ( aHR 1.21 [95% CI, 0.97–1.51])<br>Risk of lung cancer higher in NCFB group than in the non bronchiectasis group only in patients without COPD (aHR 1.19 [95% CI, 1.09–1.31]) . |

| | | | | | | | | | | |
|---|---|---|---|---|---|---|---|---|---|---|
| **Chung WS et al. (2015) [3]** | Nationwide retrospective cohort study, Taiwan | Patients enrolled in the National Health Insurance System | The incidence and risk of cancer in patients hospitalized with NCFB compared with the general population without NCFB | 4345 | 4.46 | NA | 62 (2694) | NA | NA | Higher risk of LC in NCFB pts than non bronchiectasic ones (aHR 2.40 [95% CI, 2.22–2.60]) |
| **Chung WS et al. (2016) [4]** | Nationwide retrospective cohort study, Taiwan | Patients enrolled in the National Health Insurance System | The incidence and risk of cancer in patients hospitalized with NCFB | 1210 | 4.58 | 72.8 (881)° | 67.5 (817) | NA | 35.2 (426) | Higher risk of LC in NCFB pts than non bronchiectasic ones (2.36, [95% CI = 2.19–2.55]) Higher risk of LC in NCFB population among the younger adults (aHR 5.25( [CI95%.81, 33.9] in 20–45 years) than in the older adults (aHR 5.05 [95% 3.36, 7.60] in 46–55 years, aHR 3.42 [CI95% 2.85, 4.11] in 46-65 years, and aHR 2.25(1.97, 2.57 from 65-75 years) Higher risk of LC for patients with NCFB and concomitant COPD (aHR = 1.14, 95% CI = 1.04–1.26) |
| **Kim YW et al. (2015) [5]** | Matched case–control study, Korea | COPD patients aged > 40 years from an academi | The association between NCFB and the risk of lung cancer | 10 | 1.4 | NA | NA | NA | NA | The presence of NCFB in COPD patients is inversely associated with the risk of LC (OR 0.25, 95% CI 0.12–0.52, P<0.001) The presence of NCFB is associated with a lower risk of |

| | | | | | | | | | | |
|---|---|---|---|---|---|---|---|---|---|---|
| | | c medical center | in patients with COPD | | | | | | | squamous cell carcinoma in COPD cohort (OR 0.11, 95% CI 0.03–0.49, P=0.001) |
| **Kim YW et al. (2016) [6]** | 2-centers retrospective study, Korea | Patients with NCFB aged > 40 years from two academic medical centers | The association between NCFB and the risk of lung cancer by analysing the lobar location of lung cancer in patients with underlying NCFB | 81 | NA | NA | 64.2 (52) | NA | NA | The presence of NCFB is associated with a significantly lower risk of LC in the same lobe (β-value calculated by evaluating NCFB as a risk factor of lung cancer : −1.091 , [CI95% −1.716 - 0.466, p = 0.001) |
| **Sin S et al. (2019) [7]** | Single-center retrospective cohort study, Korea | Patients aged ≥20 years who underwent chest CT drom an academic medical center | Mortality risk and causes of death between the group with NCFB and controls without lung disease | 205 | NA | NA | NA | NA | NA | Malignancy is the major cause of death in the NCFB group (31.2%), in particular LC (12.4%)<br>NCFB is significantly associated with increased LC-related death (aHR, 3.36; 95% CI, 2.18–5.18) |
| **Sanchez-Carpintero Abad et al. (2020) [8]** | Population-based cohort | Patients aged > 40 years- | Prevalence of NCFB, detection of nodules, | 5 | 6.8 | NA | NA | NA | NA | Non significant difference in incidence and prevalence of LC in NCFB population and non-bronchiectasic one |

| | | | | | | | | | | |
|---|---|---|---|---|---|---|---|---|---|---|
| | study, Spain | old, with a smoking history, without any cancer within 5 years prior to entry, from Pamplona cohort of I-ELCAP | need for additional studies and incidence of cancer in patients with NCFB | | | | | | | |

LC, lung cancer; NCFB, non-cystic fibrosis bronchiectasis; COPD, Chronic obstructive pulmonary disease; aHR, adjusted Hazard Ratio; CCI, Charlson Comorbidity Index


1. Kim, Y., K. Han, J. Yoo, H. K. Kang, T. S. Park, D. W. Park, J. Y. Hong, J. Y. Moon, S. H. Kim, T. H. Kim*, et al.* "Risk factors of incident lung cancer in patients with non-cystic fibrosis bronchiectasis: A korean population-based study." *Cancers (Basel)* 14 (2022): 10.3390/cancers14112604. https://www.ncbi.nlm.nih.gov/pubmed/35681584.
2. Choi, H., H. Y. Park, K. Han, J. Yoo, S. H. Shin, B. Yang, Y. Kim, T. S. Park, D. W. Park, J. Y. Moon*, et al.* "Non-cystic fibrosis bronchiectasis increases the risk of lung cancer independent of smoking status." *Ann Am Thorac Soc* 19 (2022): 1551-60. 10.1513/AnnalsATS.202111-1257OC. https://www.ncbi.nlm.nih.gov/pubmed/35533306.
3. Chung, W. S., C. L. Lin, C. L. Lin and C. H. Kao. "Bronchiectasis and the risk of cancer: A nationwide retrospective cohort study." *Int J Clin Pract* 69 (2015): 682-8. 10.1111/ijcp.12599. https://www.ncbi.nlm.nih.gov/pubmed/25421905.
4. Chung, W. S., C. L. Lin, W. H. Hsu and C. H. Kao. "Increased risk of lung cancer among patients with bronchiectasis: A nationwide cohort study." *QJM* 109 (2016): 17-25. 10.1093/qjmed/hcu237. https://www.ncbi.nlm.nih.gov/pubmed/25435548.
5. Kim, Y. W., K. N. Jin, E. Y. Heo, S. S. Park, H. S. Chung and D. K. Kim. "The association between combined non-cystic fibrosis bronchiectasis and lung cancer in patients with chronic obstructive lung disease." *Int J Chron Obstruct Pulmon Dis* 10 (2015): 873-9. 10.2147/COPD.S80439. https://www.ncbi.nlm.nih.gov/pubmed/26005340.
6. Kim, Y. W., C. H. Lee, K. N. Jin, J. K. Lee, E. Y. Heo, S. S. Park, H. S. Chung and D. K. Kim. "The regional association between bronchiectasis and lung cancer in chest ct." *BMC Pulm Med* 16 (2016): 151. 10.1186/s12890-016-0311-4. https://www.ncbi.nlm.nih.gov/pubmed/27846869.

7. Sin, S., S. Y. Yun, J. M. Kim, C. M. Park, J. Cho, S. M. Choi, J. Lee, Y. S. Park, S. M. Lee, C. G. Yoo*, et al.* "Mortality risk and causes of death in patients with non-cystic fibrosis bronchiectasis." *Respir Res* 20 (2019): 271. 10.1186/s12931-019-1243-3. https://www.ncbi.nlm.nih.gov/pubmed/31796019.
8. Sanchez-Carpintero Abad, M., P. Sanchez-Salcedo, J. P. de-Torres, A. B. Alcaide, L. M. Seijo, J. Pueyo, G. Bastarrika, J. J. Zulueta and A. Campo. "Prevalence and burden of bronchiectasis in a lung cancer screening program." *PLoS One* 15 (2020): e0231204. 10.1371/journal.pone.0231204. https://www.ncbi.nlm.nih.gov/pubmed/32282811.
